# Research on factors affecting serial entrepreneurial intention: An interpretive structure model

**DOI:** 10.3389/fpsyg.2022.992141

**Published:** 2022-10-25

**Authors:** Xiuwei Bai, Dejun Cheng, Yuting Chen

**Affiliations:** ^1^School of Management, Nanjing University, Nanjing, China; ^2^School of Business, Hebei Normal University for Nationalities, Hebei, China

**Keywords:** serial entrepreneurial intention, entrepreneurial reentry, interpretive structure model, entrepreneurial expectations, entrepreneurial cognitive schema, behavioral addiction tendency

## Abstract

Serial entrepreneurship is a very common phenomenon in the world. Research on serial entrepreneurs is the core of understanding entrepreneurship and entrepreneurs, such as, why entrepreneurs insist on starting businesses many times? What affects the sustainability of entrepreneurship? Based on the interpretive structure model of systems engineering, this study constructs a hierarchical model of the factors affecting serial entrepreneurial intention, which proposed the basic conditions, key factors, and paths affecting serial entrepreneurial intention. Based on this, the hierarchical model of factors affecting serial entrepreneurial intention is also tested through a typical serial entrepreneurial case. The results show that: (1) there are 16 factors affecting serial entrepreneurial intention, and each factor plays a role at a specific level; (2) entrepreneurial expectations and identification and evaluation of opportunities are the key factors affecting serial entrepreneurial intention. We can improve the ability of the identification and evaluation of opportunities through entrepreneurial failure learning, and form reasonable entrepreneurial expectations; (3) entrepreneurial cognitive schema and behavioral addiction tendency directly affect entrepreneurs’ identification and evaluation of opportunities; (4) demographic factors, financial conditions, environmental conditions, and entrepreneurial experience are the basic conditions that affect serial entrepreneurial intention indirectly through emotional perception and motivation factors.

## Introduction

Serial entrepreneurs are not limited to one entrepreneurial activity. They are representatives of entrepreneurial active groups (Vaillant and Lafuente, 2019), and the practice of serial entrepreneurship is more and more common in all countries. Furthermore, research on serial entrepreneurs is the core of understanding entrepreneurship and entrepreneurs (MacMillan, 1986), especially the sustainability of entrepreneurship. Studies have shown that serial entrepreneurs may be more likely to succeed over time (Cope, 2005) and show a more positive attitude toward entrepreneurial failure (Politis, 2008). However, when entrepreneurial activities succeed or fail, some entrepreneurs choose to end their entrepreneurial career, while others choose to start again. The intention of entrepreneurs to start again is called serial entrepreneurial intentions (Simmons et al., 2016). As Simmons asked, what are the factors that affect entrepreneurs’ choice to start again? The serial entrepreneurship intention has attracted more and more interest in the field of entrepreneurship studies.

The existing studies mainly focus on the factors affecting serial entrepreneurial intentions from three perspectives. The first one is the comparative study, which compares serial entrepreneurship with novice entrepreneurship and portfolio entrepreneurship, to obtain the characteristics of serial entrepreneurship and the factors affecting serial entrepreneurial intention. For example, the ability to recognition of opportunity is more likely to be associated with serial entrepreneurship and portfolio entrepreneurship, and higher opportunity exploration ability is associated with portfolio entrepreneurship rather than serial entrepreneurship and novice entrepreneurship (Parker, 2014). The second perspective is entrepreneurial reentry. After failure of entrepreneurship, entrepreneurs can choose to close enterprises to enter the labor market or to start a new one, and the latter will become serial entrepreneurs. At present, the research on entrepreneurial reentry mainly focuses on distress exits and failure loss, entrepreneurial learning from failure, and failure attribution (Ucbasaran et al., 2003; KoÇAk et al., 2011; Lin and Wang, 2018; Williams et al., 2020). The last one is the antecedent variables affecting serial entrepreneurial intention, mainly including the characteristics of entrepreneurs, traits and entrepreneurial experience, and so on (Plehn-Dujowich, 2009; Spivack et al., 2014; Hsu et al., 2017b; Simmons et al., 2018; Williams et al., 2020). To sum up, the factors affecting serial entrepreneurial intention are complex, and the achievements of relevant research are rich. However, due to the relatively scattered perspectives, the internal structure of how the complex factors affect serial entrepreneurial intention is unclear, so a definite hierarchical model needs to be established.

This study has sorted out 36 factors that may affect serial entrepreneurial intention based on the literature review. After the analysis and discussion of the expert group, 16 factors are finally formed. Then, using the method of interpretive structure model, the hierarchical structure is obtained, which proposes the mutual relationship and multilevel structure of the factors affecting serial entrepreneurial intention. Moreover, this study further tests the hierarchical structure model of factors affecting serial entrepreneurial intention through a case study.

## Literature review

Serial entrepreneurs are more likely to run their businesses more successfully. Success may make entrepreneurs fall into the trap of complacency and perform poorly in subsequent entrepreneurship, whereas if failed entrepreneurs can bear the “sadness” that may prevent them from returning to entrepreneurship, they will learn from failure and improve themselves (Shepherd, 2003). Entrepreneurs with failed entrepreneurial experience are 17% less likely to restart a business than entrepreneurs with successful entrepreneurial experience (Amaral et al., 2011). Existing studies show that the factors influencing serial entrepreneurial intention can be categorized into 16 individual factors, 12 entrepreneurial level factors, and eight environmental factors as presented in Table 1.

**TABLE 1:** Identified factors of serial entrepreneurial intention.

Notation	Factors	Type of research	Relationship	References
**Individual factors**				
A1	Sensation-seeking trait disposition	Quantitative	Positive	Carr et al., 2016
A2	Workaholism trait disposition	Quantitative	Positive	Carr et al., 2016
A3	behavioral addiction to entrepreneurship	Qualitative	Related	Spivack et al., 2014
A4	Age	Quantitative	Negative	Lin and Wang, 2018
A5	Gender	Quantitative	Related	Shinnar et al., 2012; Antoncic et al., 2015; Simmons et al., 2018
A6	Failure loss	Quantitative	Positive/negative	Lin and Wang, 2018
A7	Perceived financial gains (prior venture)	Quantitative	Negative	Hsu et al., 2017b
A8	Performance feedback from prior business	Quantitative	Negative	Carr et al., 2016
A9	Career stage	Quantitative	Inverted U shaped	Baù et al., 2017
A10	Emotional intensity and emotional valence	Qualitative	Related	Williams et al., 2020
A11	Positive/negative emotion	Qualitative	Related	Williams et al., 2020
A12	Risk aversion	Conceptual	Related	Parker, 2014
A13	Anxiety	Quantitative	Related	Zelekha et al., 2018
A14	Confidence	Qualitative	Related	Hayward et al., 2010
A15	Entrepreneurial self-efficacy	Quantitative	Positive	Antoncic et al., 2016; Hsu et al., 2017b; Antoncic et al., 2021
A16	Prevention focused cognition	Quantitative	Negative	Simmons et al., 2016
**Entrepreneurial factors**				
A17	The length of venture creation experience	Quantitative	Positive	Hsu et al., 2017a
A18	Entrepreneurial failure	Quantitative	Positive/negative	Lafuente et al., 2018; Tian and Cao, 2021
A19	Entrepreneurial success	Quantitative	Positive	Amaral et al., 2011
A20	Harvest exits/Distress exits	Quantitative	Positive/negative	Simmons et al., 2016
A21	Expectation of new venture’s prospects and existing business	Qualitative	Related	KoÇAk et al., 2011
A22	Entrepreneurial learning from failure	Quantitative	Positive	KoÇAk et al., 2011; Tian and Cao, 2021
A23	Entrepreneurial cognitive schema	Quantitative and deductive	Related	Vaillant and Lafuente, 2019
A24	Opportunity identification	Conceptual	Related	Parker, 2014
A25	Opportunity evaluation	Qualitative	Related	KoÇAk et al., 2011; Carbonara et al., 2019
A26	Entrepreneurial experience	Quantitative and deductive	Positive	Stam et al., 2008; Vaillant and Lafuente, 2019
A27	Entrepreneurial skill	Conceptual	Related	Plehn-Dujowich, 2009
A28	Failure attributions	Quantitative/qualitative	Related	Ucbasaran et al., 2003; Williams et al., 2020
**Environmental factors**				
A29	Relational capital	Qualitative	Related	KoÇAk et al., 2011
A30	Structural capital	Qualitative	Related	KoÇAk et al., 2011
A31	Social capital (family/friend support)	Quantitative	Positive	Stam et al., 2008; Lin and Wang, 2018
A32	Bankruptcy laws	Quantitative	Related	Lee et al., 2011
A33	Stigma of entrepreneurial failure	Quantitative	Related	Simmons et al., 2013
A34	Visibility of information about prior failures	Quantitative	Related	Simmons et al., 2013
A35	Labor market rigidity	Quantitative	Positive	Fu et al., 2018
A36	Market volatility	Quantitative	Negative	Zhang and Wang, 2020

### Individual factors

The individual factors affecting serial entrepreneurial intention are mainly studied from two perspectives. First of all, personal traits. Some studies have pointed out that both Sensation-seeking trait disposition (A1) and workaholism trait disposition (A2) will affect serial entrepreneurship (Carr et al., 2016); The psychological, emotional, and physiological aspects of entrepreneurial experience strengthen the behavioral addiction to entrepreneurship (A3), which will promote individuals to repeatedly carry out entrepreneurial activities (Spivack et al., 2014). In addition, age and gender are also important factors affecting serial entrepreneurship. The older the entrepreneur’s age (A4), the slower the speed of restarting (Lin and Wang, 2018). Career stages (A9) are related to the possibility of entrepreneurs’ reentry after failure, the relationship of which is inverted U shaped (Baù et al., 2017). Moreover, males score higher than females on openness factor which may be the most important factor of the big five personality, which differentiates entrepreneurs from other people (Antoncic et al., 2015). Gender moderates the negative relationship between the perceived lack of support barrier and the entrepreneurial intention, which exposes some cross-cultural differences, and that females (relative to males) perceive the lack of support barrier, fear of failure, and lack of competency barriers as more important in entrepreneurial activities (Shinnar et al., 2012). Probability of female entrepreneurs (A5) returning to entrepreneurial activities after failure is less than that of males (Simmons et al., 2018).

Individual psychological perception is another perspective from which many scholars also put forward the factors affecting serial entrepreneurial intention. Some studies have pointed out that the perceived failure loss (A6) has a slightly significant negative impact on the speed of entrepreneurial reentry (Lin and Wang, 2018), however, when the perceived failure loss is very huge, entrepreneurs may be motivated by failure to reenter into entrepreneurial activities (McGrath, 1999). The more individuals actively describe their entrepreneurial experience according to perceived financial gains (A7) or losses from their prior venture, the weaker their subsequent entrepreneurial intention is, and vice versa (Hsu et al., 2017b). The individuals who receive positive performance feedback (A8) from prior ventures have strong serial entrepreneurial intentions (Carr et al., 2016). At the same time, studies have shown that individual emotions also have a significant impact on serial entrepreneurial intention. Negative emotion (A11) is not necessarily an obstacle to reentry into entrepreneurial activities as previously thought, the interaction between controllability and emotion is the core of explaining entrepreneurial reentry (Williams et al., 2020). Moreover, it is further found that the interaction between failure attribution and emotional intensity/emotional valence (A10) will affect the way of individual entrepreneurial reentry (Williams et al., 2020). Entrepreneurs with high-risk aversion are more likely to be novice entrepreneurs, while entrepreneurs with low-risk aversion (A12) are more likely to be serial entrepreneurs (Parker, 2014). Entrepreneurial failure will make entrepreneurs anxious (A13). The higher degree of anxiety, the greater the tendency of a person to become a salaried employee after the first entrepreneurial failure. The less anxious he is, the more inclined he is to regard entrepreneurship as a way of life and adhere to it in entrepreneurial behavior (Zelekha et al., 2018). In addition, scholars have pointed out that entrepreneurs’ psychological capital is one of the factors affecting serial entrepreneurship intention. Entrepreneurs with more confidence (A14) can better recover from emotional, cognitive, social, and economic ventures, and are more likely to conduct subsequent ventures (Hayward et al., 2010). As the dimensions of entrepreneurial self-efficacy, financial self-efficacy and marketing self-efficacy are related to entrepreneurial intention. Family business environment may be very important for individuals to develop financial self-efficacy, which affects entrepreneurial intention (Antoncic et al., 2021), and that individuals with higher marketing self-efficacy are more likely to create a firm (Antoncic et al., 2016). Under the same conditions, the higher the entrepreneurial self-efficacy (A15), the higher the subsequent entrepreneurial intention. Moreover, the degree of entrepreneurial self-efficacy will moderate the impact of financial loss after entrepreneurial failure on subsequent entrepreneurial intentions (Hsu et al., 2017b). There is also a significant negative correlation between prevention-focused cognition (A16), which is one of the regulatory focuses of entrepreneurs, and serial entrepreneurial intention (Simmons et al., 2016).

### Entrepreneurial level factors

The entrepreneurial level factors may directly affect the serial entrepreneurial intention. Firstly, many studies have shown that entrepreneurial experiences can affect the serial entrepreneurial intention, such as the length of venture creation experience (A17), experienced entrepreneurial failure (A18) (Hsu et al., 2017a; Lafuente et al., 2018). However, domestic scholars also proposed that entrepreneurial failure has a positive impact on serial entrepreneurial intention (Tian and Cao, 2021). Entrepreneurs with entrepreneurial success (A19) are more likely to reenter faster (Amaral et al., 2011). Exit mode is an important factor affecting serial entrepreneurship intentions. If entrepreneurs are the prevention focus, distress exits (A20) reduce the serial entrepreneurial intention of such entrepreneurs (Simmons et al., 2016). In addition, entrepreneurs’ expectations of new venture’s prospects (A21) or current business can affect the motivation of entrepreneurs, which provides incentives for entrepreneurs to reenter entrepreneurial activities (KoÇAk et al., 2011).

Second, entrepreneurial cognition is also an emphasized factor affecting serial entrepreneurial intention. Domestic scholars put forward that entrepreneurial failure affects the willingness to start a business again through entrepreneurs’ learning from failure (A22) (Tian and Cao, 2021), which has been proved to be the “entrepreneurial catalyst” to entrepreneurial reentry (KoÇAk et al., 2011). The learning process generated from past entrepreneurial experiences may affect the entrepreneurial cognitive schema (A23), which may be important for the decision to set up a new company (Vaillant and Lafuente, 2019). Moreover, opportunity identification (A24) and opportunity appraisal (A25) are the key factors affecting entrepreneurs to become a serial entrepreneur, which provide the inducement to reenter into entrepreneurial activities (KoÇAk et al., 2011; Parker, 2014; Carbonara et al., 2019). Studies also proposed that failure attribution (A28) is one of the factors affecting the way to effectively reenter into entrepreneurship after failure (Williams et al., 2020), and that those entrepreneurs who attribute success to internal factors will become habitual entrepreneurs (Ucbasaran et al., 2003). Furthermore, attribution to internal and controllable factors has a significant positive impact on their serial entrepreneurial intention (Zhu et al., 2021).

Third, existing studies focus on the human capital affecting serial entrepreneurship intention (Carbonara et al., 2019). Relevant studies have further verified that human capital seems to be positively associated with the revival of entrepreneurship, in which entrepreneurial experience (A26) has the strongest impact, and the second is the general human capital (Stam et al., 2008). The past entrepreneurial experience, whether positive or negative, will significantly affect the entrepreneurial reentry (Vaillant and Lafuente, 2019). An entrepreneur with high entrepreneurial skills (A27) will continue to operate if he has enough profits. When the expectation of existing venture’s prospects is negative, he will choose to become a serial entrepreneur (Plehn-Dujowich, 2009).

### Environmental factors

Environmental factors are also important factors affecting serial entrepreneurial intention. First of all, the social capital of entrepreneurs. Studies have shown that the strong or weak relationship in structural capital (A30) plays a crucial role in the process of entrepreneurial reentry. Strong relationship can support entrepreneurs from exit to reentry, while weak relationship plays a key role in recognizing and taking advantage of new opportunities. Meanwhile, relational capital (A29) in the form of trust has great benefits in the process of entrepreneurial reentry and can promote interpersonal relations and subsequent business transactions (KoÇAk et al., 2011). Entrepreneurs with family or friend support (A31) seem to adhere to their preference for entrepreneurship without being intimidated by negative entrepreneurial events (Stam et al., 2008). Although family support can provide multiple resources and psychological support for serial entrepreneurs and help entrepreneurs recover from negative entrepreneurial events, the impact of family support on serial entrepreneurial intention is not direct, but mixed (Lin and Wang, 2018).

Second, there are legal factors affecting serial entrepreneurial intention. Studies have shown that a friendly bankruptcy law (A32) can reduce barriers to reentry, which means less time and less cost, and give entrepreneurs a new start by encouraging them to take more risks and set up more new companies (Lee et al., 2011).

Third, social factors can also affect serial entrepreneurial intention. In the environment with low visibility of information about prior failures (A34) and high public stigma of entrepreneurial failure (A33), failed entrepreneurs are more likely to engage in entrepreneurial activities again (Simmons et al., 2013). Some studies have proposed that the labor market rigidity (A35) increases the possibility of individuals’ reentry into entrepreneurial activities, and market volatility (A36) also affects the relationship between entrepreneurial learning from failure and serial entrepreneurial intention (Zhang and Wang, 2020).

To sum up, according to the literature review, there are 16 factors affecting serial entrepreneurial intention after categorization of 36 factors as given in Table 2.

**TABLE 2:** Identified factors of serial entrepreneurial intention.

Notation	Critical factors	Descriptive definition	Category
F1	Behavioral addiction tendency	The tendency to seek out a feeling or action intensely and continuously. (A1, A2, A3)	Individual factor
F2	Demographic factors	Demography, age and gender. (A4, A5)	Individual factor
F3	Financial conditions	The economic performance of entrepreneurship. (A6, A7, A8)	Individual factor
F4	Social capital	The intangible resources that entrepreneurs derive from their position in the social structure, such as trust, support, and social networks. (A29, A30, A31)	Environmental factor
F5	Entrepreneurship experience	Entrepreneur has been undergone in entrepreneurial activity, length of the startup, success or fail. (A17, A18, A19, A20)	Entrepreneurial factor
F6	Entrepreneurial expectation	Entrepreneurs’ expectations for the future development of current entrepreneurial or future entrepreneurial activities. (A21)	Entrepreneurial factor
F7	Emotion perception	Entrepreneurs perceive their emotion as positive or negative, or anxious. (A10, A11, A13)	Individual factor
F8	Psychological capital	The positive psychological state of the entrepreneur which provide the psychological resources to promote performance. (A14, A15)	Individual factor
F9	Entrepreneurial learning from failure	Entrepreneurs learn from the entrepreneurial failure. (A22)	Entrepreneurial factor
F10	Career stage	The career stage of the entrepreneur, early, middle and late. (A9)	Individual factor
F11	Human capital	knowledge, skills, abilities, etc. of an entrepreneur. (A26, A27)	Entrepreneurial factor
F12	Environment conditions	Environmental factors which effect entrepreneurship, including economy, government, social culture and laws. (A32, A33, A34, A35, A36)	Environmental factor
F13	Entrepreneurial cognitive schema	The cognitive structures developed in entrepreneurship which deal with different entrepreneurial situation. (A23)	Entrepreneurial factor
F14	Opportunity identification and evaluation	Entrepreneurs identify and evaluate opportunities in the entrepreneurship. (A24, A25)	Entrepreneurial factor
F15	Failure attributions	Entrepreneurs consider the reason which lead to entrepreneurial failure. (A28)	Entrepreneurial factor
F16	Motivation factors	Internal motivations and dynamics that regulate or influence entrepreneurial behavior. (A16, A12)	Individual factor

## Materials and methods

### Methods

Interpretative structural model (ISM) is a kind of structure modeling technique, which was developed by Professor Warfield to analyze the problems related to complex social and economic systems (Warfield, 1978; Muruganantham et al., 2018). The ISM refers to a process that transforms unclear and poorly articulated models of systems into visible and well-defined models (Sushil, 2012). This method decomposes the complex system into several sub-system elements, extracts the interaction mechanism between the elements of the complex system with practical experience and knowledge, and finally formed a theoretical construct (Valmohammadi and Dashti, 2016). Compared with the traditional empirical analysis method of influencing factors, the ISM method is characterized by dynamically supplementing the required data according to the research progress. Given its advantages in dynamicity, complementarity and integrity, ISM method has been applied to many studies in the field of management, such as human resource, entrepreneurship, and engineering management (Mandal and Deshmukh, 1994; Wei et al., 2019; Liu et al., 2021).

The main concepts involved in the paper include general matrix, adjacency matrix, reachability matrix, and the highest-level element set. A general matrix is a rectangular table with m rows and n columns composed of i × j numbers, and the element a_*ij*_ represents the element in row i and column j.

The adjacency matrix describes the direct relationship between each row and column of factors. For the general system S (F_1_, F_2_,…, F_*n*_) with n factors, the adjacency matrix is defined as A = [a_*ij*_]n × n, where a_*ij*_ = 1 (when element F_*i*_ has a direct effect on F_*j*_) or 0 (when elements F_*i*_ have no direct effect on F_*j*_).

The reachable matrix is used to represent the direct or indirect relationship between the influencing factors. Using the operational properties of Boolean matrices, the reachable matrix R satisfies the equation: (A + I)^*k*–1^≠(A + I)*^k^* = (A + I)^*k*+1^ = R, where A represents the adjacency matrix, I represents the identity matrix, and K represents the number of operations.

Highest-level element set refers to a set of elements that cannot reach other elements except themselves. R(F_*i*_) refers to the reachable set of F_*i*_ and C(F_*j*_) represents the antecedent set of F_*j*_. If R(F_*i*_) = R(F_*i*_)∩C(F_*j*_) (where i = j), R(F_*i*_) is placed in a set corresponding to the level and excluded in the analysis of subsequent levels, then R(F_*i*_) is the highest-level element set (Hussain et al., 2016).

This paper uses ISM method to carry out a study on the factors influencing serial entrepreneurial intention, including four steps. This paper firstly identifies the antecedent factors of SEI through literature review. Secondly, an expert group is set up to screen out the important factors from the antecedent factors and determine the relationship between the factors. Thirdly, using statistical software (e.g., MATLAB), we design the relationship structure of each factor and obtain the corresponding reachability matrix. Fourthly, this research carries out hierarchical processing and forms a multilevel conceptual model based on the reachability matrix.

### Analysis

Existing literature has studied the antecedents of serial entrepreneurial intention from multiple perspectives. We firstly identify 16 factors (as shown in Table 2) through literature review to help further screen by the expert panel.

In the second step, an expert panel was established to clarify the key factors affecting SEI and interrelation of 16 factors. The panel consists of seven members, including two scholars in the research field of entrepreneurship, three serial entrepreneurs, and two doctoral students. After all the members of the expert panel understand the basic concepts of SEI and the 16 antecedents, they further judged back-to-back whether the 16 factors had an important impact on SEI. The result of the discussion showed that 16 factors were unanimously agreed by more than four members (Kuo et al., 2010).

Thirdly, the relationship between 16 factors was discussed and seven members of the expert panel were asked to conduct a pair-wise comparison of 16 factors. We denoted the 16 factors as F_*i*_, where i = 1, 2, ……, 16, as given in Table 2. The experts were asked to select from one of the following four types when judging the relationship between the factor F_*i*_ and F_*j*_:

• Type V: factor F_*i*_ influences factor F_*j*_ directly
• Type A: factor F_*j*_ influences factor F_*i*_ directly
• Type X: factor F_*i*_ influences factor F_*j*_ each other
• Type O: factor F_*i*_ and factor F_*j*_ are mutually unrelated

In the process of judging the relationship between factors, we still adopt the opinions of most experts (more than four members), and the final relationship between the 16 elements presented is unanimously confirmed by all the members, as presented in Table 3.

**TABLE 3:** Pair-wise comparison of 16 factors.

The type of the relationship between factors F_i_ and F_j_															Critical factors
O	O	V	O	O	O	O	O	O	O	O	X	O	O	A	Behavioral addiction tendency (F_1_)
V	V	V	V	O	V	O	V	V	V	O	V	O	V	Demographic factors (F_2_)	
V	O	O	O	O	O	O	V	O	V	V	O	O	Financial conditions (F_3_)		
O	O	V	O	O	O	O	O	V	O	O	X	Social capital (F_4_)			
O	O	V	V	O	V	O	V	V	V	V	Entrepreneurship experiences (F_5_)				
A	O	A	A	A	A	O	O	A	O	Entrepreneurial expectation (F_6_)					
O	O	V	O	O	O	O	V	O	Emotion perception (F_7_)						
O	O	O	O	O	O	O	X	Psychological capital (F_8_)							
O	A	O	X	O	V	O	Entrepreneurial learning from failure (F_9_)								
O	O	O	V	O	V	Career stage (F_10_)									
O	V	V	V	O	Human capital (F_11_)										
V	O	V	O	Environment conditions (F_12_)											
O	V	V	Entrepreneurial cognitive schema (F_13_)												
A	A	Opportunity identification and evaluation (F_14_)													
A	Failure attributions (F_15_)														
Motivation factors (F_16_)															

In the fourth step, we used matrix operations to divide the 16 important influencing factors into different levels and thus get a multilevel ISM. A 16 × 16 square matrix was used to express the logical correlation among the important factors affecting SEI based on Table 3, forming an adjacency matrix A that covers any two or two elements in the whole influencing factors system. In this matrix, a_*ij*_ refers to the elements in line i and column j of a square matrix (i, j = 1, 2, ……, 16), indicating the relationship between factors F_*i*_ and F_*j*_. “0” in row i and column j represents that factor i has no direct influence on the factor j, while “1” indicates factor i directly influences factor j. The results expressed in 16 × 16 adjacency matrix from Table 3 are presented in Table 4.

**TABLE 4:** Adjacency matrix A of 16 factors.

No	F_1_	F_2_	F_3_	F_4_	F_5_	F_6_	F_7_	F_8_	F_9_	F_10_	F_11_	F_12_	F_13_	F_14_	F_15_	F_16_
F_1_	1	1	0	0	1	0	0	0	0	0	0	0	0	0	0	0
F_2_	0	1	0	0	0	0	0	0	0	0	0	0	0	0	0	0
F_3_	0	1	1	0	0	0	0	0	0	0	0	0	0	0	0	0
F_4_	0	0	0	1	1	0	0	0	0	0	0	0	0	0	0	0
F_5_	1	1	0	1	1	0	0	0	0	0	0	0	0	0	0	0
F_6_	0	0	1	0	1	1	0	1	0	0	1	1	1	1	0	1
F_7_	0	1	1	0	1	0	1	0	0	0	0	0	0	0	0	0
F_8_	0	1	0	1	1	0	0	1	1	0	0	0	0	0	0	0
F_9_	0	1	1	0	1	0	1	1	1	0	0	0	1	0	1	0
F_10_	0	0	0	0	0	0	0	0	0	1	0	0	0	0	0	0
F_11_	0	1	0	0	1	0	0	0	1	1	1	0	0	0	0	0
F_12_	0	0	0	0	0	0	0	0	0	0	0	1	0	0	0	0
F_13_	0	1	0	0	1	0	0	0	1	1	1	0	1	0	0	0
F_14_	1	1	0	1	1	0	1	0	0	0	1	1	1	1	1	1
F_15_	0	1	0	0	0	0	0	0	0	0	1	0	1	0	1	1
F_16_	0	1	1	0	0	0	0	0	0	0	0	1	0	0	0	1

As the influencing factors of complex systems are not directly related, we use the reachability matrix (R) to obtain the relationship between the direct and indirect effects of one factor on other factors, as well as the transitive representation of each factor. In order to express the transfer relationship between the direct or indirect effects of 16 factors, we need to convert adjacency matrix into reachable matrix.

Element r_*i*_ can reach r_*j*_ by the distance of unit 1, and r_*j*_ can still reach the next influencing factor by the distance of unit 1 in the reachability matrix. We add adjacent matrix A and unit matrix I to get matrix B, which can further get the reachability matrix through Boolean algebraic power operation with the help of software MATLAB. According to the operation rules of transforming adjacent matrix into reachable matrix, we calculate Bn until the calculation satisfies B^*k*–1^ = B*^k^* (K = 15), which shows direct and indirect relationships among 16 influencing factors of SEI, as is presented in Table 5.

**TABLE 5:** Reachability matrix R of 16 factors.

No	F_1_	F_2_	F_3_	F_4_	F_5_	F_6_	F_7_	F_8_	F_9_	F_10_	F_11_	F_12_	F_13_	F_14_	F_15_	F_16_
F_1_	**1**	0	0	1	1	1	1	1	1	0	1	0	1	1	1	0
F_2_	1	**1**	1	1	1	1	1	1	1	0	1	0	1	1	1	1
F_3_	0	0	**1**	0	0	1	1	1	1	0	1	0	1	1	1	1
F_4_	1	0	0	**1**	1	1	1	1	1	0	1	0	1	1	1	0
F_5_	1	0	0	1	**1**	1	1	1	1	0	1	0	1	1	1	0
F_6_	0	0	0	0	0	**1**	0	0	0	0	0	0	0	0	0	0
F_7_	0	0	0	0	0	1	**1**	1	1	0	1	0	1	1	1	0
F_8_	0	0	0	0	0	1	0	**1**	1	0	1	0	1	1	1	0
F_9_	0	0	0	0	0	1	0	1	**1**	0	1	0	1	1	1	0
F_10_	0	0	0	0	0	1	0	1	1	**1**	1	0	1	1	1	0
F_11_	0	0	0	0	0	1	0	1	1	0	**1**	0	1	1	1	0
F_12_	0	0	0	0	0	1	0	1	1	0	1	**1**	1	1	1	1
F_13_	0	0	0	0	0	1	0	1	1	0	1	0	**1**	1	1	0
F_14_	0	0	0	0	0	1	0	0	0	0	0	0	0	**1**	0	0
F_15_	0	0	0	0	0	1	0	1	1	0	1	0	1	1	**1**	0
F_16_	0	0	0	0	0	1	0	1	1	0	1	0	1	1	1	**1**

Bold values represent the correlation between each element and itself is 1.

## Results

Based on reachability matrix, this paper sorts out the highest-level element set. When R(Fi) = R(Fi)∩C(Fi), R(Fi) is placed in a set corresponding to the level and excluded in the analysis of subsequent levels. This paper continues to find the new highest-level elements from the remaining reachability matrix, and then finds the highest-level elements contained in each level by analogy. For example, after the first hierarchical process, the element satisfies R(F_*i*_) = R(F_*i*_)∩C(F_*i*_) is 6, so {6} is the first level. After that, 14 is found to satisfy the condition after the element containing 6 is removed from the list, so 14 is the second layer. In the same way, this paper divides these 16 factors into six levels, and the final multilevel structure hierarchy is presented in Table 6. The final hierarchical results were obtained as follows:

**TABLE 6:** Interpretive structure model analysis of 16 factors.

Level	R(Fi)	C(Fi)	R(F_i_) ∩ C(F_j_)
1	1, 4, 5, 6, 7, 8, 9, 11, 13, 14, 15	1, 2, 4, 5	1, 4, 5
	1, 2, 3, 4, 5, 6, 7, 8, 9, 11, 13, 14, 15, 16	2	2
	3, 6, 7, 8, 9, 11, 13, 14, 15, 16	2, 3	3
	1, 4, 5, 6, 7, 8, 9, 11, 13, 14, 15	1, 2, 4, 5	1, 4, 5
	1, 4, 5, 6, 7, 8, 9, 11, 13, 14, 15	1, 2, 4, 5	1, 4, 5
	6	1, 2, 3, 4, 5, 6, 7, 8, 9, 10, 11, 12, 13, 14, 15, 16	**6**
	6, 7, 8, 9, 11, 13, 14, 15	1, 2, 3, 4, 5, 7	7
	6, 8, 9, 11, 13, 14, 15	1, 2, 3, 4, 5, 7, 8, 9, 10, 11, 12, 13, 15, 16	8, 9, 11, 13, 15
	6, 8, 9, 11, 13, 14, 15	1, 2, 3, 4, 5, 7, 8, 9, 10, 11, 12, 13, 15, 16	8, 9, 11, 13, 15
	6, 8, 9, 10, 11, 13, 14, 15	10	10
	6, 8, 9, 11, 13, 14, 15	1, 2, 3, 4, 5, 7, 8, 9, 10, 11, 12, 13, 15, 16	8, 9, 11, 13, 15
	6, 8, 9, 11, 12, 13, 14, 15, 16	12	12
	6, 8, 9, 11, 13, 14, 15	1, 2, 3, 4, 5, 7, 8, 9, 10, 11, 12, 13, 15, 16	8, 9, 11, 13, 15
	6, 14	1, 2, 3, 4, 5, 7, 8, 9, 10, 11, 12, 13, 14, 15, 16	14
	6, 8, 9, 11, 13, 14, 15	1, 2, 3, 4, 5, 7, 8, 9, 10, 11, 12, 13, 15, 16	8, 9, 11, 13, 15
	6, 8, 9, 11, 13, 14, 15, 16	2, 3, 12, 16	16

2	1, 4, 5, 7, 8, 9, 11, 13, 14, 15	1, 2, 4, 5	1, 4, 5
	1, 2, 3, 4, 5, 7, 8, 9, 11, 13, 14, 15, 16	2	2
	3, 7, 8, 9, 11, 13, 14, 15, 16	2, 3	3
	1, 4, 5, 7, 8, 9, 11, 13, 14, 15	1, 2, 4, 5	1, 4, 5
	1, 4, 5, 7, 8, 9, 11, 13, 14, 15	1, 2, 4, 5	1, 4, 5
	7, 8, 9, 11, 13, 14, 15	1, 2, 3, 4, 5, 7	7
	8, 9, 11, 13, 14, 15	1, 2, 3, 4, 5, 7, 8, 9, 10, 11, 12, 13, 15, 16	8, 9, 11, 13, 15
	8, 9, 11, 13, 14, 15	1, 2, 3, 4, 5, 7, 8, 9, 10, 11, 12, 13, 15, 16	8, 9, 11, 13, 15
	8, 9, 10, 11, 13, 14, 15	10	10
	8, 9, 11, 13, 14, 15	1, 2, 3, 4, 5, 7, 8, 9, 10, 11, 12, 13, 15, 16	8, 9, 11, 13, 15
	8, 9, 11, 12, 13, 14, 15, 16	12	12
	8, 9, 11, 13, 14, 15	1, 2, 3, 4, 5, 7, 8, 9, 10, 11, 12, 13, 15, 16	8, 9, 11, 13, 15
	14	1, 2, 3, 4, 5, 7, 8, 9, 10, 11, 12, 13, 14, 15, 16	**14**
	8, 9, 11, 13, 14, 15	1, 2, 3, 4, 5, 7, 8, 9, 10, 11, 12, 13, 15, 16	8, 9, 11, 13, 15
	8, 9, 11, 13, 14, 15, 16	2, 3, 12, 16	16

3	1, 4, 5, 7, 8, 9, 11, 13, 15	1, 2, 4, 5	1, 4, 5
	1, 2, 3, 4, 5, 7, 8, 9, 11, 13, 15, 16	2	2
	3, 7, 8, 9, 11, 13, 15, 16	2, 3	3
	1, 4, 5, 7, 8, 9, 11, 13, 15	1, 2, 4, 5	1, 4, 5
	1, 4, 5, 7, 8, 9, 11, 13, 15	1, 2, 4, 5	1, 4, 5
	7, 8, 9, 11, 13, 15	1, 2, 3, 4, 5, 7	7
	8, 9, 11, 13, 15	1, 2, 3, 4, 5, 7, 8, 9, 10, 11, 12, 13, 15, 16	**8, 9, 11, 13, 15**
	8, 9, 11, 13, 15	1, 2, 3, 4, 5, 7, 8, 9, 10, 11, 12, 13, 15, 16	**8, 9, 11, 13, 15**
	8, 9, 10, 11, 13, 15	10	10
	8, 9, 11, 13, 15	1, 2, 3, 4, 5, 7, 8, 9, 10, 11, 12, 13, 15, 16	**8, 9, 11, 13, 15**
	8, 9, 11, 12, 13, 15, 16	12	12
	8, 9, 11, 13, 15	1, 2, 3, 4, 5, 7, 8, 9, 10, 11, 12, 13, 15, 16	**8, 9, 11, 13, 15**
	8, 9, 11, 13, 15	1, 2, 3, 4, 5, 7, 8, 9, 10, 11, 12, 13, 15, 16	**8, 9, 11, 13, 15**
	8, 9, 11, 13, 15, 16	2, 3, 12, 16	16

4	1, 4, 5, 7	1, 2, 4, 5	1, 4, 5
	1, 2, 3, 4, 5, 7, 16	2	2
	3, 7, 16	2, 3	3
	1, 4, 5, 7	1, 2, 4, 5	1, 4, 5
	1, 4, 5, 7	1, 2, 4, 5	1, 4, 5
	7	1, 2, 3, 4, 5, 7	**7**
	10	10	**10**
	12, 16	12	12
	16	2, 3, 12, 16	**16**

5	1, 4, 5	1, 2, 4, 5	**1, 4, 5**
	1, 2, 3, 4, 5	2	2
	3	2, 3	**3**
	1, 4, 5	1, 2, 4, 5	**1, 4, 5**
	1, 4, 5	1, 2, 4, 5	**1, 4, 5**
	12	12	**12**

6	2	2	**2**

Bold values represent the factors of each level in the interpretative structural model.

• Level 1: 6
• Level 2: 14
• Level 3: 8, 9, 11, 13, 15
• Level 4: 7, 10, 16
• Level 5: 1, 3, 4, 5, 12
• Level 6: 2

**FIGURE 1:**
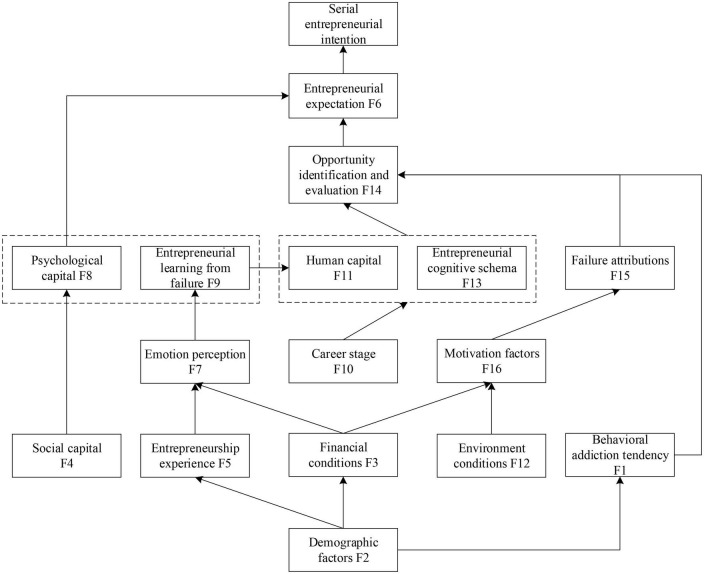
Interpretive structure model.

Based on the reachability matrix and highest-level element sets, the multi-level structure hierarchy chart of serial entrepreneurship intention is drawn, from which interpretive structure model of key factors affecting serial entrepreneurship intention is obtained (as shown in Figure 1). According to the figure, factors affecting serial entrepreneurship intention show a multilevel hierarchical structure with six levels. The specific analysis is summarized as follows:

Entrepreneurial expectation is the key factor affecting serial entrepreneurial intention, which directly affects serial entrepreneurial intention. The research shows that entrepreneurs’ psychological capital has an indirect impact on serial entrepreneurial intention, and entrepreneurial expectation is the mediator. Entrepreneur can cultivate entrepreneurs’ psychological capital such as self-confidence and entrepreneurial self-efficacy through a variety of social support methods; meanwhile, entrepreneurial learning from failure helps to promote entrepreneurs’ ability of opportunity identification and evaluation, which affects entrepreneurial expectations. Therefore, entrepreneurs should be encouraged to learn from failure and improve their human capital.

Opportunity identification and evaluation play an important role in the formation of serial entrepreneurial intention. Entrepreneurial cognitive schema and behavioral addiction tendency have a direct impact on opportunity identification and evaluation. At different career stages, the entrepreneurial cognitive schema shows differentiated characteristics. Entrepreneurs can enrich entrepreneurial cognitive schema through continuous learning over time. Behavioral addiction tendency has been found as an important driving factor, which is mainly affected by demographic factors.

In addition, this study also shows that demographic factors, financial conditions, environment conditions, and entrepreneurship experience are the basic conditions affecting serial entrepreneurial intention, which indirectly affect serial entrepreneurial intention, and emotional perception and motivation factors are the mediators. To be specific, entrepreneurial experience and financial conditions directly affect entrepreneurs’ emotional perception. Emotional regulation is closely related to how to learn after entrepreneurial failure. The environment conditions and financial conditions will directly affect the motivation of entrepreneurs and indirectly affect the failure attribution.

## Case study

This research chooses a case of serial entrepreneur to study the factors affecting serial entrepreneurship intention in order to test the model we have obtained above. There are three main reasons for choosing this case. First, the entrepreneur in this case has the intention to start a new business after the success or failure. Second, he is a typical serial entrepreneur with many entrepreneurial experiences. Third, it is convenient to obtain data about this case. The entrepreneur in this case is a typical representative of Chinese internet entrepreneurs, which easily provides a large number of data. Therefore, this case is representative for studying the factors affecting serial entrepreneurship intention. Based on the principle of true and valid data selection and sources, we collected news interviews, published books, company materials, and publicly published academic research results related to serial entrepreneurial behavior to ensure the effectiveness of case analysis.

The entrepreneur in the case, represented as *A*, has started businesses for six times, all in the internet industry. After many entrepreneurial failures, the internet service company he founded has been listed, which ranks third in Internet industry of China with a market value of more than 140 billion dollars now. Entrepreneurial process can be roughly divided into three stages (Wang, 2020).

In the first stage, *A* and his partners formed an entrepreneurial team to start their business in China. They established three social networking sites in succession because of the belief that social networking sites (opportunity identification and evaluation) were a valuable and promising opportunity (entrepreneurial expectation). Although they focused on the internet industry, which they were familiar with and identified valuable opportunities, all the three startups failed. *A* noted that the previous two startups failed because of the emphasis on products and the neglect of promotion (entrepreneurial learning from failure), so they paid more attention to promotion (human capital) in the third startup. From this, we can see that learning from failure promote human capital, which laid the foundation for the next startup. However, the financial return of the third social networking site was not satisfactory (entrepreneurial expectation), resulting in being sold. This failure was so hard for him that he chose employment for a while. As mentioned above, *A* started businesses for three times in succession because of the good entrepreneurial expectations for the identified opportunities and voluntarily sold the third social networking sites because of the bad expectation for financial return, which interpreted that entrepreneurial expectation is the key factor affecting serial entrepreneurial intention, and that identification and evaluation of opportunity indirectly affect serial entrepreneurship intention through entrepreneurship expectation.

In the second stage, *A* chose to start a new business again after 1 year’s employment. *A* looked for opportunities which were promising and focused on the blog after much thought (opportunity identification and evaluation). He built two blog websites in succession in this stage, but he still failed for various reasons. The first blog website was going well at first, but it had to be shut down because of unexpected accident that a large number of sensitive remarks caused by irregular management appeared in blogs. However, the entrepreneurial failure did not make *A* lose his confidence but showed his maturity to the entrepreneurial team which strengthened the confidence of the entrepreneurial team (psychological capital), and built the second blog website at last. From this, we can see that psychological capital affects serial entrepreneurial intention. As mentioned above, *A* started businesses five times and focused on an Internet-related entrepreneurship program in the first and second stages, which reflected that he was very persistent in starting businesses and actively looked for entrepreneurial opportunities in the Internet industry (opportunity identification and evaluation). As *A* said in the interview: “I don’t regard entrepreneurship itself as a special thing. It’s just my lifestyle and I have an extreme adherence to entrepreneurship” (behavioral addiction tendency). So behavioral addiction tendency affects serial entrepreneurial intention through opportunity identification and evaluation.

In the third stage, *A* constantly studied websites and products and finally found an entrepreneurial opportunity, that is a business website (opportunity identification and evaluation). He determined the path to build a business website based on the experience of previous failures and development mode of internet marketing he summarized (entrepreneurial cognitive schema). He established a group-buying website and achieved great performance. As mentioned above, entrepreneurial cognitive schema affects serial entrepreneurial intention through opportunity identification and evaluation. *China Youth Daily* once published *A*’s words: The entrepreneurs failed because of immature opportunity which was incompatible with the environment 10 years ago. However, it does not mean that this thing should not be done, and it may be successful to do it at another time (entrepreneurial environment), showing that the entrepreneurial environment is a basic factor affecting serial entrepreneurship intention.

To sum up, the discussion of the case is in line with the interpretive structure model constructed in this paper, the key factors which are affecting serial entrepreneurship intention show hierarchical characteristics.

## Conclusion

This study sorted out 16 key factors affecting serial entrepreneurship intention. According to the method of interpretive structure model of system engineering, this study constructs a hierarchical model of the factors affecting serial entrepreneurial intention and tests it through case study, which defines the key factors, basic conditions, and paths affecting serial entrepreneurial.

The results of this study show that entrepreneurial expectation is the key factor affecting serial entrepreneurial intention, which directly affects serial entrepreneurial intention. Entrepreneurs may be forced to quit the enterprise due to insolvency, or they may take the initiative to quit the existing enterprise because the performance of the enterprise fails to meet the expectations of entrepreneurs (Westhead et al., 2005; Ucbasaran et al., 2010), or because they find new business opportunities (Hessels et al., 2011). When entrepreneurs’ distress exits, they can improve their ability to identify and evaluate opportunities through entrepreneurial recovery and learning from failure, which will help them to form reasonable entrepreneurial expectations. While they choose to exit, they may have serial entrepreneurial intention due to their positive expectation of new business opportunities. Moreover, the exit mode will also affect entrepreneurial expectation through the entrepreneur’s psychological capital. Different from previous studies that focus on the distress exits affecting serial entrepreneurial intention, this study believes that the formation of serial entrepreneurial intention of entrepreneurs who take the initiative to quit is also a topic that needs to be paid attention to. In addition, this study proposes that social capital has a significant impact on entrepreneurs’ psychological capital. The risk and pressure of entrepreneurship are alleviated by social support, which helps to stimulate entrepreneurial resilience of entrepreneurs (Zhang and Li, 2020) and improve their psychological resilience, finally affecting serial entrepreneurial intention.

This study also shows that identification and evaluation of opportunity play an important role in the formation of serial entrepreneurial intention. Entrepreneurial cognitive schema and behavioral addiction tendency have a direct effect on identification and evaluation of opportunity, which in turn affects serial entrepreneurial intention. Vaillant and Lafuente (2019) proposed that the learning process generated in the past entrepreneurial experience may affect entrepreneurial cognitive schema, which is very important for an entrepreneur to reenter into new entrepreneurship and become a serial entrepreneur. The findings of this study not only further explain the path of entrepreneurial experience affecting serial entrepreneurial intention but also indicate that there is a cognitive mechanism behind opportunity identification. In addition, entrepreneurs who have the behavioral addiction tendency will think compulsively and look for innovation and opportunities continuously (Spivack et al., 2014), to become serial entrepreneurs. Furthermore, demographic factors directly affect behavioral addiction tendency, which indicates that behavioral addiction tendency is related to physiological factors to a certain extent.

In addition, demographic factors, financial conditions, environmental conditions, and entrepreneurial experience are the basic conditions that affect serial entrepreneurial intention, which work indirectly mainly through emotional perception and motivation factors. The findings in this study help us better understand the persistence of entrepreneurial spirit and analyze the formation process of serial entrepreneurial intention.

## Discussion

### Implications

This study has three main aspects in theoretical contribution. Firstly, the ISM model clearly interprets the internal relationship and hierarchical structure of the factors affecting serial entrepreneurial intention and makes contributions to understanding serial entrepreneurship intention in depth. Although the existing literature has integrated the factors affecting serial entrepreneurship intention (Tipu, 2020), which has not constructed the internal relationship and hierarchical structure of the influencing factors. As Zhao et al. (2014) note that the studies on the factors affecting serial entrepreneurship intention still lack depth, and what role the factors play and how the factors exert their influence need to be further analyzed. Based on the existing literature on the factors affecting serial entrepreneurship intention, this study constructs an ISM model showing a multilevel hierarchical structure with six levels, which defines the key factors, basic conditions, and paths affecting serial entrepreneurial intention. Secondly, this study contributes to the theoretical development of serial entrepreneurship research. The results of this study show that entrepreneurial expectation is the key factor affecting serial entrepreneurial intention, which directly affects the serial entrepreneurial intention, and that identification and evaluation of opportunity indirectly affect serial entrepreneurship intention through entrepreneurship expectation. As Parker (2014) notes, the key factor that decides why some people become serial entrepreneurs while others remain novice entrepreneurs is the identification and evaluation of opportunity, and identification and evaluation of opportunity play an important role in the formation of serial entrepreneurial intention. This is proved by this study. Furthermore, this study puts forward influencing mechanism of identification and evaluation of opportunity, which further supplements the conclusion and defines the key role played by entrepreneurial expectation. In addition, from results, we also suggest that entrepreneurial cognitive schema and behavioral addiction tendency have a direct effect on identification and evaluation of opportunity, which provides new perspectives and useful clues for opportunity cognition mechanism. Existing studies have identified the unique regular pattern of identification and evaluation of opportunities of serial entrepreneurs, but have not yet explored the cognitive mechanism behind the regular pattern (Yu et al., 2020). Prototype model is one of the recognition modes for entrepreneurs to find opportunities, the higher the matching degree between things and prototypes, the more likely they are to find entrepreneurial opportunities (Shane, 2003). Entrepreneurial cognitive schema affects the prototypes and cognitive modes of opportunity identification, which is a useful clue. At the same time, behavioral addiction tendency as a special pathological feature is closely related to the individual nervous system and can affect individual cognition (Moore et al., 2021), which provides a new perspective for the study of the mechanism of opportunity cognition. Moreover, entrepreneurs with behavioral addiction tendency will have such special behaviors as compulsive thinking, conceit, and neglect of family and friends (Spivack et al., 2014), which reflects the dark side of entrepreneurial activities. This study finds that demographic factors directly affect behavioral addiction tendency, which provides useful clues for the study of the dark side of entrepreneurial activities. Thirdly, the ISM model shows multiple influencing paths of the factors affecting serial entrepreneurial intention, which provides a framework for the research of serial entrepreneurship intention. Although some influencing paths have been confirmed by empirical research (Parker, 2014; Wang et al., 2018; Zhang and Wang, 2020), some paths still need to be explored. It is interesting to note that in Figure 1, individual factors and environmental factors are below the third level, and the entrepreneurial factors are above the fourth level except for entrepreneurial experience (F5). Based on the role and the descriptive definition of entrepreneurial experience in ISM model, it is found that more attention is paid to the entrepreneurial failure context in existing studies and the research on the mode of distress exits exit is more extensive, while the research on the mode of taking the initiative to exit is lacking, which may be one of the reasons why the entrepreneurial factors are above the fourth level except for entrepreneurial experience (F5).

The conclusion of this study provides enlightenment for entrepreneurs and entrepreneurial management organizations in managing entrepreneurial activities. Firstly, entrepreneurial expectation is the direct key factor affecting serial entrepreneurship intention. Psychological capital affects entrepreneurial expectation, which in turn affects serial entrepreneurial intention. Entrepreneurial self-efficacy affects the willingness to participate in entrepreneurial activities in the future (Hsu et al., 2017b). Maintaining a high degree of self-efficacy in entrepreneurial activities can enhance serial entrepreneurship intention and make entrepreneurs more persistent. Although self-confidence helps to recover from entrepreneurial failure, entrepreneurs’ overconfidence in environmental cognition will reduce entrepreneurial performance (Li and Cheng, 2018), so entrepreneurs should maintain moderate and reasonable self-confidence and high entrepreneurial self-efficacy. Secondly, identification and evaluation of opportunity have a direct effect on entrepreneurial expectation, which in turn affects serial entrepreneurial intention. So entrepreneurs should effectively improve their ability to identify and evaluate opportunities. This study also proposes two strategies to improve the ability of identification and evaluation of opportunity. One, human capital (e.g., rich entrepreneurial experience) helps entrepreneurs to identify entrepreneurial opportunities and strengthen their ability to evaluate and develop entrepreneurial opportunities (Ucbasaran et al., 2003, 2008). Entrepreneurs should effectively learn and absorb entrepreneurial failure experience, especially in the context of entrepreneurial failure. Two, entrepreneurs enrich their entrepreneurial cognitive schema through continuous learning (Vaillant and Lafuente, 2019), providing an effective cognitive mechanism for the identification and evaluation of opportunities. Thirdly, financial conditions and entrepreneurial environment are the basic conditions affecting serial entrepreneurship intention. Therefore, government departments should provide entrepreneurial education such as failure education and emotional education to guarantee the entrepreneurs’ learning. At the same time, the government needs to provide strong support in entrepreneurship policy, both financially and psychologically, to create a good economic and social environment for entrepreneurial activities.

### Limitation and future research

This study provides some new ideas and directions for future research, but there are still some limitations. Firstly, this study uses the method of interpretive structure model to propose a hierarchical structure model of the factors affecting serial entrepreneurial intention. The method is one method of systems engineering that has been partly applied in the field of entrepreneurship (Muruganantham et al., 2018; Wei et al., 2019), but the applicability of it still needs to be further studied. Secondly, the hierarchical model proposed in this study lacks strong empirical support. Future research can carry out empirical exploration of relevant approaches to provide empirical support for the relationship between factors. Finally, the single case study in this paper seems not enough to fully explain the interpretive structure model of factors affecting serial entrepreneurial intention. As Cardon et al. (2011) note approaches of sense-making under different cultural backgrounds may have different effects on individual intentions and behaviors. Future research can enrich the model using more cases from different cultural backgrounds.

In addition, the hierarchical model proposed in this study provides a new research approach and direction for future research on serial entrepreneurial intention. First of all, we need to pay more attention to the research on exit modes, especially the mode of taking the initiative to exit. As indicated earlier, entrepreneurs may actively quit entrepreneurship or passively quit entrepreneurship, but less attention is paid to the mode of taking the initiative to exit in existing studies (Yu et al., 2020). Along this line, we need to further explore the influencing mechanism of different exit modes on serial entrepreneurship intention, especially the impact of the mode of taking the initiative to exit on subsequent entrepreneurial decisions. Secondly, we need to continue to explore the cognitive mechanism behind entrepreneurs’ opportunity identification. Entrepreneurs use the cognitive structure of identifying opportunities to compare new ideas with opportunities, to identify opportunities (Santos et al., 2015). Entrepreneurial cognitive schema is an “action-based knowledge structure” used by entrepreneurs based on highly developed and orderly knowledge (Mitchell et al., 2000), so entrepreneurs identify opportunities that match the prototypes in entrepreneurial cognitive schema. However, this study shows that serial entrepreneurs enrich their entrepreneurial cognitive schema through continuous learning, which can update the cognitive structure used to identify opportunities. Whether entrepreneurs only recognize the opportunities that match the prototypes or update their entrepreneurial cognitive schema (prototype model) to identify opportunities still needs further exploration. At the same time, the pathological perspective is a new perspective for the study of cognitive mechanism behind entrepreneurs’ opportunity identification. As Moore et al. (2021) note the pathological characteristics of entrepreneurs affect their cognitive structure, so we need to continue to explore the impact of other types of neurological or pathological characteristics on opportunity recognition, such as insomnia and obsessive-compulsive personality disorder. Thirdly, we need to further explore the dark side of entrepreneurship. Emotional reaction, performance feedback, and entrepreneur–enterprise connection in entrepreneurial activities will all become reinforcing factors of behavioral addiction to entrepreneurship (Yu et al., 2021). Along this line, future research can continue to explore reinforcing factors of behavioral addictive tendency, such as physiological factors and other dark sides of entrepreneurial activities.

## Data availability statement

The original contributions presented in this study are included in the article/supplementary material, and the datasets generated for this study are available on request to the corresponding author.

## Author contributions

XB was responsible for drafting the manuscript, as well as the acquisition, analysis and interpretation of data. DC and YC participated in the data analysis and revising of the manuscript. All authors have read and approved the final manuscript.
